# Immune escape of SARS-CoV-2 variants to therapeutic monoclonal antibodies: a system review and meta-analysis

**DOI:** 10.1186/s12985-023-01977-5

**Published:** 2023-11-15

**Authors:** Huichun Shi, Jiajia Sun, Yigang Zeng, Xiaomeng Wang, Shanshan Liu, Lijun Zhang, Enming Shao

**Affiliations:** 1 Shanghai Public Health Clinical Center, Fudan University, Shanghai, 201508 China; 2 The First Affiliated Hospital of Zhengzhou University, Zhengzhou University, Zhengzhou, 450099 China

**Keywords:** SARS-CoV-2 Omicron, Vaccines-induced antibodies, Neutralization

## Abstract

**Background:**

Omicron's high transmissibility and variability present new difficulties for COVID-19 vaccination prevention and therapy. In this article, we analyzed the sensitivity of vaccine-induced antibodies as well as the effect of booster vaccinations against Omicron sublineages.

**Methods:**

We looked for Randomized Controlled Trials and cohort studies that reported the COVID-19 vaccines against Omicron sublineages up to 28 July 2022 through PubMed, the Cochrane Library, EMBASE, and Web of Science. Quantitative synthesis was carried out using Stata 16.0 and RevMa5.3, then the serum NT50 and antibody sensitivity to neutralize Omicron sublineages were assessed before and after booster vaccination. This study was registered with PROSPERO number CRD42022350477.

**Results:**

This meta-analysis included 2138 patients from 20 studies, and the booster vaccination against Omicron sublineages showed a significant difference compared to 2 dosage: BA.1/BA.1.1 (SMD = 0.80, 95% CI: 0.75–0.85, *P* = 0.00), BA.2/BA.2.12.1 (SMD = 0.77, 95% CI: 0.69–0.85, *P* = 0.00), BA.3 (SMD = 0.91, 95% CI: 0.83–1.0, *P* = 0.00), and BA.4/5 (SMD = 0.77, 95% CI: 0.60–0.94, *P* = 0.00). The sensitivity of vaccines-induced antibodies decreased by at least 5-folds after booster vaccination, particularly in the case of BA.4/5 which had the most notable decline in vaccine effectiveness.

**Conclusion:**

After the booster vaccination, the NT50 and the neutralization ability of vaccine-induced antibodies increased, but the susceptibility of antibodies decreased compared with the control virus, which may be a clue for future Omicron sublineages prevention.

**Supplementary Information:**

The online version contains supplementary material available at 10.1186/s12985-023-01977-5.

## Introduction

At the end of 2019, SAR-CoV-2 broke out and immediately started a pandemic over the world. According to estimates, more than 570 million individuals have contracted the disease, and more than 6.4 million have passed away (https://covid19.who.int/). The RNA virus SARS-CoV-2 is constantly changing its genome [1]. Most variants have minimal impact, while a few may evolve into the dominant strain through natural selection [2, 3]. Omicron (B.1.1.529) was initially endemic to BA.1 infection, which contains large number of mutations, with more than 30 spike proteins (including receptor binding domains (RBD) and N-terminal domains), mainly clustered at the interaction sites of strong neutralizing antibodies, such as sites that interact with ACE2 and NTDs, etc. [4, 5]. These mutations not only lead to an increase in transmissibility, but also to a significant reduction in the neutralization titer of the fraction of serum that is immune to natural and vaccine induction, leading to lots of vaccine breakthrough infections [1, 6, 7].

Subsequently, BA.2 beat BA.1 and began to rise rapidly around the world. Compared to the Omicron BA.1 variant, BA.2 has four unique substitutions which 1 reversion (S446G) and 3 new added mutations (T376A, D405N, and R408S), together with an alternative mutation (S371L replaced by S371F in BA.1) in the RBD [8, 9]. It has been reported that BA.2 has a 1.5-fold higher transmission rate than BA.1, but the reason is still not clear, which may be related to immune evasion caused by new mutations (https://covid.cdc.gov/). Not only that, vaccine-induced antibodies from donors who received ChAdOx1 and BNT162b2 vaccines showed a certain reduction in neutralization titers against Omicron sublineages, and the effectiveness of vaccines also was limited to 0–8.8% [8, 10]. Afterwards, it was found that a novel mutant BA.2.12.1 had a similar RBD sequence to BA.2 but included L452Q [11, 12]. Later, BA.3 was discovered, but it is currently quite uncommon [13, 14]. The BA.4/BA5 mutation appears to have a growth advantage over BA.1 and BA.2, therefore it is more prevalent and able to escape immune from vaccine-induced antibodies [15]. Besides, due to the addition of L452R/F486V mutants, BA.4 and BA.5 have a greater transmission advantage over BA.2 [15]. Now, increasing numbers of BA.4 and BA.5 infections are being reported globally [16], thus there is an urgent need to study the receptor binding and immune evasion capabilities of these new Omicron sublineages. Although Omicron's RBD introduces multiple mutations, both the BNT162b2 (Pfizer-biontech) and the mRNA-1273 (Moderna) vaccines still can produce high titers of Omicron-neutralizing antibodies regarding the durability of this response [17]. Considering that the current understanding of the effects of homologous and heterologous vaccines is not comprehensive, some studies have been conducted to assess the strength and short-term persistence of the neutralizing activity of booster vaccines against omicron [18].

Increased infectivity or virulence of emerging omicron subsets further highlights the importance of vaccination programs and effective public health measures, hence comprehensive evidence is urgently needed to validate the vaccine effectiveness of COVID-19 vaccines against Omicron subsets and susceptibility to serum antibodies after vaccination. Although several studies have evaluated the vaccine efficacy of vaccine-induced antibodies and therapeutic antibodies against SARS-CoV-2 VOCs [19, 20], some recent findings have not been included, and new SASR-CoV-2 omicron sublines are continuing to emerge. Therefore, to gain insight into the impact of COVID-19 vaccines on Omicron sublineages, we conducted a comprehensive systematic review and meta-analysis, including randomized controlled trials and cohort studies, to investigate the effectiveness and immune escape of Omicron sublineages after booster vaccination.

## Methods

### Data sources and search

This research protocol was registered on PROSPERO (CRD42018088882), which followed Preferred Reporting Items for Systematic reviews and Meta-Analyses (PRISMA) guidelines with no changes, we searched for literatures published on PubMed, Embase, Web of Scicence, and Cochrane Library using the search terms “COVID-19”, “SARS-CoV-2”, “Omicron”, and “BA.1 OR BA.1.1 OR BA.2 OR BA.2.12.1 OR BA.4/5” and “Vaccines” to search, the detailed search strategy was shown in the Supplementary Material (Search Strategy). This searches were last updated on 28 July 2022.

### Selection of studies

The effectiveness and NT50 (concentration for 50% of neutralization titer) of COVID-19 vaccines against Omicron sublineages, including BA.1, BA.1.1 OR BA.2, BA.2.12.1, BA.3, and BA.4/5, were evaluated in randomised controlled trials (RCTs) and cohort studies. We included the general population over the age of 18 who had Omicron sublineages infection and following exceptions: (1) research protocols, reviews, news, animal studies; (2) patients with other diseases or special populations (such as healthcare workers); (3) antibodies or antiviral drug neutralization research. The major data was the VE of full vaccination against Omicron sublineanges (NT50 exceeds threshold after booster vaccination) and the NT50 after booster immunization. Searches are restricted to English-language papers.

### Data extraction and quality assessment

Two researchers independently reviewed titles/abstracts and collected information on included studies (such as first author, year of publication, study type, country, subject characteristics, vaccine type, etc.), if there was any disagreement between the researchers, a third researcher was consulted. The methodological quality and risk of bias in RCTs were assessed using the Cochrane Collaborative Risk of Bias Assessment tool, and the risk of bias in cohort study was assessed using the Newcastle-Ottawa Scale (NOS).

### Statistical analysis

In our paper, Stata version 16.0 and Review Mananger (RevMan) 5.3 were used for Meta-merged analysis. Continuous variable data were expressed as standardized mean difference (SMD) and 95% Confidence Interval (95%CI) were used as effect indicators, and I^2^ and *P* tests were used for heterogeneity test. The fixed-effect model was employed if *P* < 0.05, I^2^ ≤ 50% indicated that the included literature was homogeneous; *P* < 0.05, I^2^ > 50% indicated that the included literature was heterogeneous; then the random-effects model was applied. *P* ≤ 0.05 was used to determine whether the combined effect was significant.

## Results

### Literature search and study characteristics

**Fig. 1:**
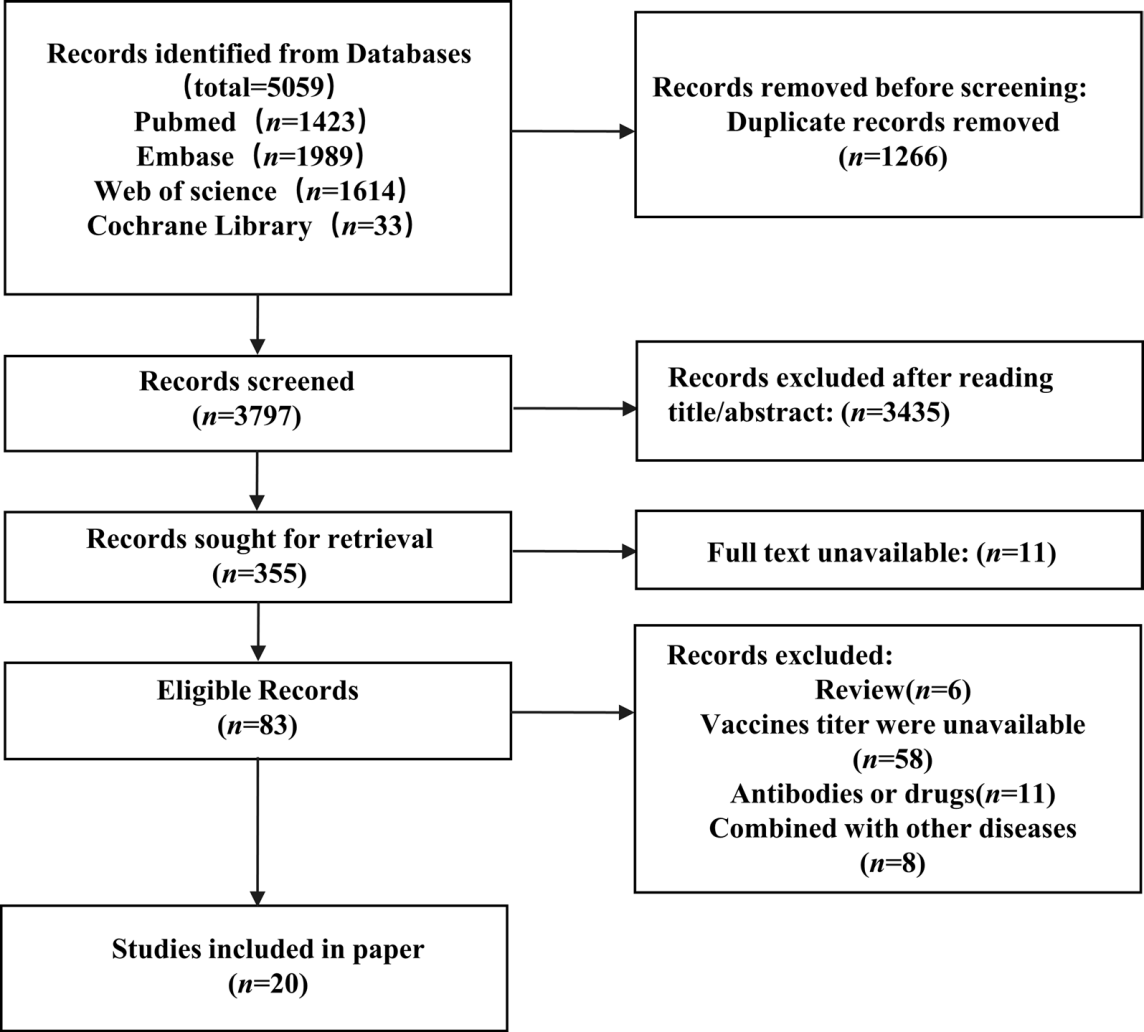
Flow chart showing the screening process for included studies

The database yielded 5059 studies in total, of which 1266 duplicates were eliminated using EndNote X9. 355 papers remained after reading titles and abstracts, while 344 papers were available for full text. After secondary screening of the obtained papers, 83 papers remained, and 20 papers were included after reading the full text, of which 6 studies were RCTs and 13 studies were cohort study. The details of the search process were presented in Fig. 1.

A total of 20 studies met the inclusion criteria with 2138 patients. All studies were published in 2022. Patients were vaccinated with 2 or 3 dosages of COVID-19 vaccines, and serum testing was performed for Omicron neutralization titers, detail characteristics of eligible studies were showed in Table 1.

**Table 1:** Study characteristics and participants demographics

Authors	Study design	Age	Vaccines	Dose	Variants	Control virus	Mean DPV	Country	Participates(*n*)
Arora [3]	Cohort study	37(27–60)	BNT162b2	3 doses	BA.1, BA.2, BA.3	B.1	21	Germany	30
Bowen [21]	Cohort study	47.5(35–79)	BNT 162b2, mRNA-1273, AZD1222, Sputnik	2/3 doses	BA.1, BA.2	D614G	29	USA	89
Cheng [2]	RCT	51.2(22–76)	BNT162b2, CoronaVac	2/3 doses	BA.1	Wild Type	28	Hongkong	116
Evans [4]	RCT	36(25–61)	BNT 162b2, mRNA-1273	2/3 doses	BA.1, BA.1.1, BA.2	D614G	21–28	USA	20
Hachmann [22]	Cohort study	35(23–76)	BNT162b2	2/3dose	BA.1, BA.2, BA.2.12.1, BA4/5	Wild Type	29	USA	27
Kawaoka [23]	RCT	> 18	BNT 162b2, mRNA-1273	2/3 doses	BA.1, BA.1.1	Wild Type	30	Japan	44
Kurhade [13]	Cohort study	55(22–74)	BNT 162b2	3 doses	BA.1, BA.2, BA.3	Wild Type	30	USA	92
Kurhade_2 [14]	Cohort study	55(22–74)	BNT162b2	3 doses	BA.1, BA.2, BA.2.12.1, BA.3, BA4/5	Wild Type	30	USA	132
Lyke [18]	Cohort study	50(25–55)	mRNA-1273	2/3 doses	BA.1, BA.2, BA.2.12.1, BA.3, BA4/5	D614G	29	USA	96
Park [24]	Cohort study	> 18	BNT162b2	2/3 doses	BA.1, BA.2, BA.5	D614G	50	USA	10
Pedersen [25]	Cohort study	57(50–60)	BNT162b2	2/3 doses	BA.1, BA.2	Wild Type	35	Denmark	64
Qu_1 [26]	Cohort study	35(23–76)	BNT 162b2, mRNA-1273	2/3 doses	BA.1, BA.2, BA.2.12.1, BA4/5	D614G	21–28	USA	60
Qu_2 [27]	Cohort study	35(26–61)	BNT 162b2, mRNA-1273	2/3 doses	BA.2.12.1, BA.4/5	D614G	21	USA	112
Sablerolles [28]	RCT	41(30–50)	Ad26.COV2.S, BNT 162b2, mRNA-1273	3 doses	BA.1	Wild Type	28	USA	279
Tuekprakhon [5]	Cohort study	> 18	BNT 162b2, mRNA-1273, AZD1222	3 doses	BA.1, BA.11, BA.2, BA.3, BA4/5	B.1	28	UK	300
Willett_1 [29]	RCT	< 40	ChAdOx1, BNT162b2, mRNA-1273	2/3 doses	BA.1	B.1	14	UK	48
Willett_2 [30]	RCT	50–90	ChAdOx1, BNT162b2, mRNA-1273	2/3 doses	BA.1, BA.2, BA4/5	Wild Type	17–28	UK	84
Yu [31]	Cohort study	34(23–69)	BNT162b2	2/3 doses	BA.1, BA.2	Wild Type	14	Israel	72
Zhou_1 [8]	Cohort study	41(32–50)	mRNA	2/3 doses	BA.1, BA.2	D614G	/	USA	14
Zhou_2 [32]	Cohort study	41.6(20–64)	BNT162b2, ChAdOx1	2/3 doses	BA.1, BA.1.1, BA.2	D614G	30	Hongkong	449

1. The reference is indicated by the first author’s surname followed by the year of publication, *n* number of participants. 2. Randomized Controlled Trial (RCT); Days-post vaccinated (DPV)

### Risk of bias

Six studies were RCTs, and the risk of bias assessment revealed that four of them "Missing outcome data" and "Deviations from intended interventions" were unclear risks, other studies had low risk of bias, finally, three were rated as low risk of bias and three were rated as moderate risk of bias (Additional file 1: Table S1, Figures S1 and S2). While 14 cohort studies were evaluated based on the risk score, 4 were of moderate quality and 10 were of high quality (Additional file 1: Table S2). All studies had a reasonable level of quality and the meta-findings analyses remained consistent.

### Vaccine effectiveness of COVID-19 vaccines against Omicron sublineages

#### VE effect on Omicron BA.1/BA.1.1

**Fig. 2:**
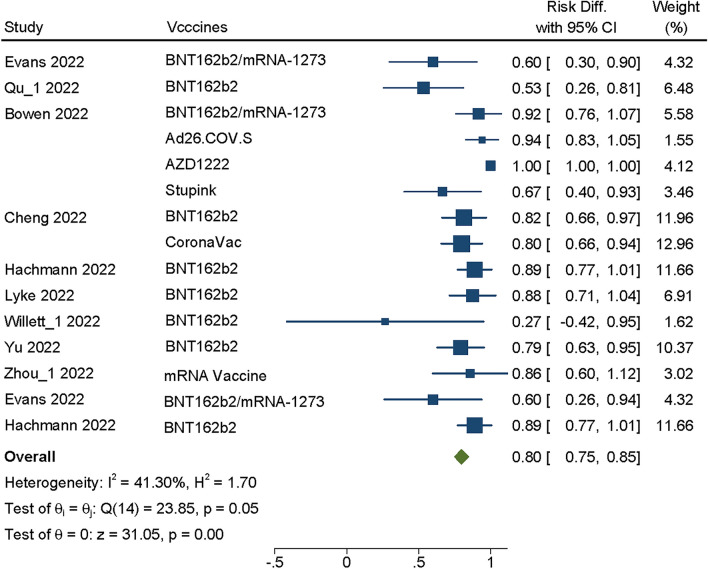
Forest plot about VE of COVID-19 vaccines against BA.1/BA.1.1. Analyses were performed using fixed effect model, the SMD and 95%CI for the EV of booster dose were displaying in figure. EV was effectiveness of vaccine; CI was confidence interval

A total of 15 vaccines from 9 studies [2, 4, 8, 18, 21, 22, 26, 29, 31] were included in the analysis to analyze the neutralizing titers of Omicron BA.1/BA1.1 in vaccine-induced antibodies after the booster doseage of COVID-19 vaccines. The heterogeneity test of these studies showed *P* = 0.05, I^2^ = 41.3%, the fixed effects model was used. The summary VE of booster vaccination against the BA.1/BA.1.1 increased compared with control virus with SMD = 0.80, 95% CI(0.75–0.85), *P* = 0.00 (Fig. 2), the difference was statistically significant(*P* > 0.05), this result indicates that even though BA.1/BA.1.1 is reported to be immune escape, booster vaccination still has a certain protective effect on patients, which is consistent with some reported results [25, 33].

#### VE effect on Omicron BA.2/BA.2.12.1

**Fig. 3:**
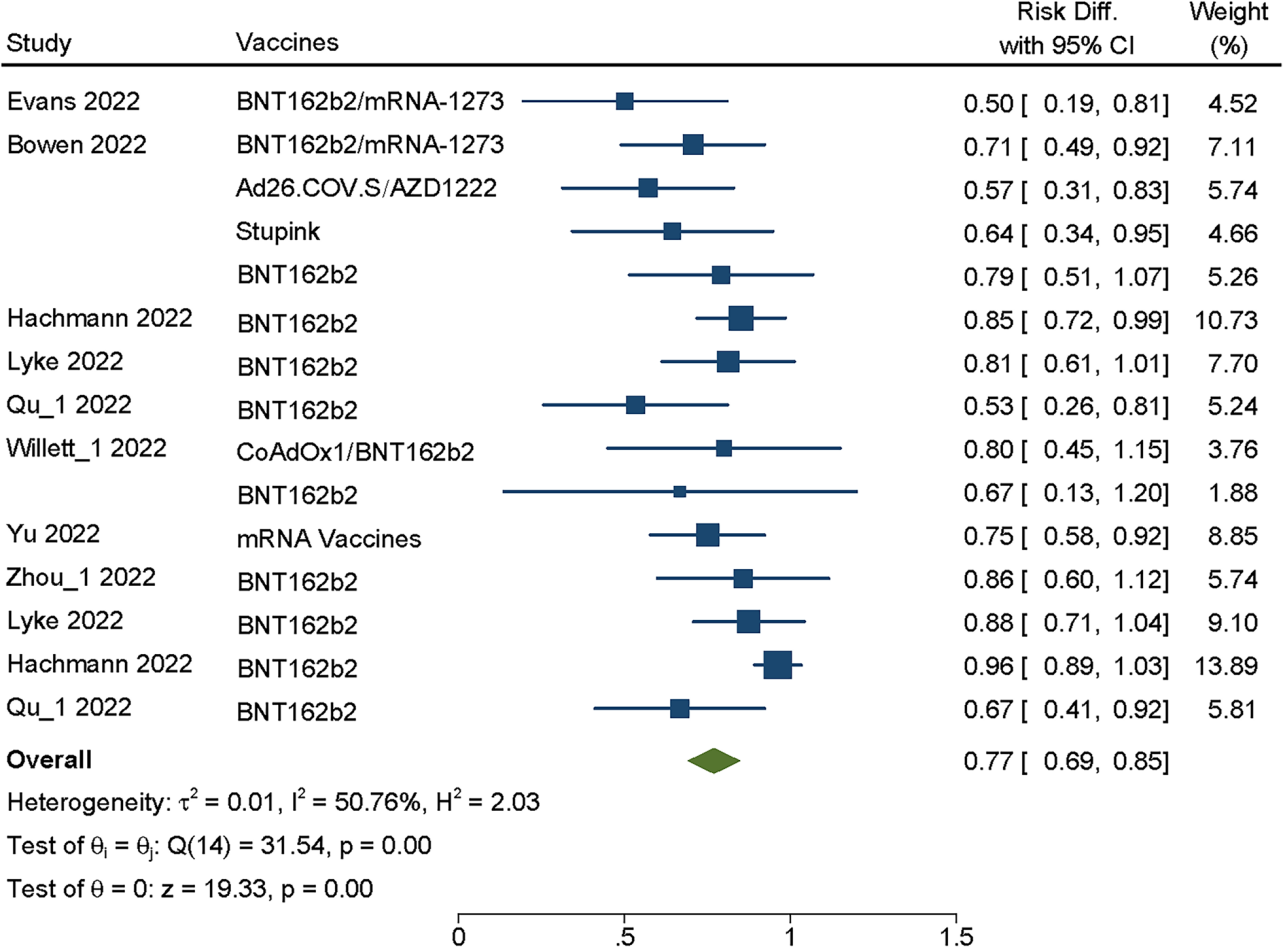
Forest plot about VE of COVID-19 vaccines against BA.2/BA.2.12.1. Analyses were performed using random effect model, the SMD and 95%CI for the EV of booster dose were displaying in figure. EV was effectiveness of vaccine; CI was confidence interval

Fifteen vaccines in 7 studies [4, 18, 21, 22, 26, 29, 31] reported the neutralization titers of BA.2/BA.2.12.1 after booster dose of COVID-19 vaccine, among which BNT162b2 was the most widely used. The heterogeneity test among these studies showed *P* = 0.00, I^2^ = 50.76%, then fixed effects model was used to analyze the summary VE. Finally, we found that the summary VE of booster vaccination against the BA.2/BA.2.12.1 increased dramatically compared with control virus with SMD = 0.77, 95% CI(0.69–0.85), *P* = 0.00 (Fig. 3), the difference was statistically significant (*P* > 0.05). It was clear that the vaccine would still neutralize BA.2/BA.2.12.1 and has a protective effect on human body.

#### VE effect on Omicron BA.3

**Fig. 4:**
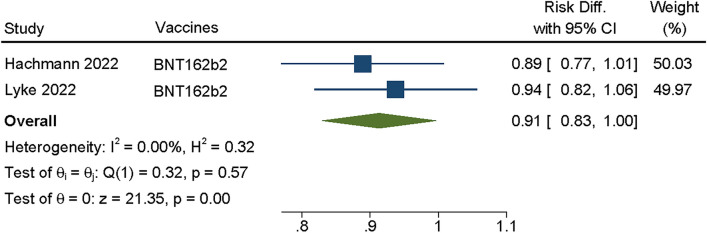
Forest plot about VE of COVID-19 vaccines against BA.3. Analyses were performed using random effect model, the SMD and 95%CI for the EV of booster dose were displaying in figure. EV was effectiveness of vaccine; CI was confidence interval

At present, the frequency of BA.3 is still relatively low [13], and there are few studies about BA.3, so only two articles were included in our research [18, 22]. We conducted meta-analysis on the two articles, and fixed effects model was used for *P* = 0.57, I^2^ = 0.00%.It is showed that the summary VE of booster vaccination against the BA.3 increased dramatically compared with control virus with SMD = 0.91, 95% CI(0.83,1.0), *P* = 0.00 (Fig. 4), the difference was statistically significant(*P* > 0.05). However, considering the relatively small sample size, reliable conclusions still need to be further increased sample size for analysis.

#### VE effect on Omicron BA.4/5

**Fig. 5:**
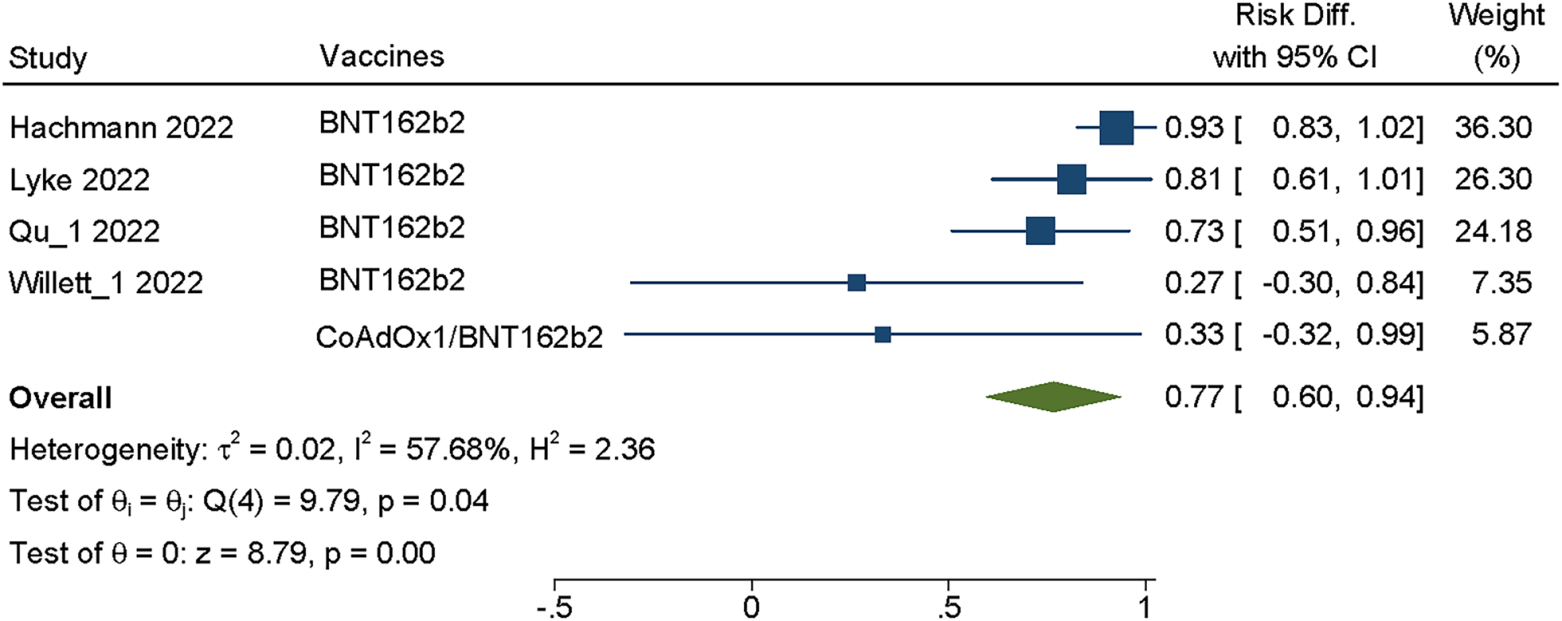
Forest plot about VE of COVID-19 vaccines against BA.4/5. Analyses were performed using random effect model, the SMD and 95%CI for the EV of booster dose were displaying in figure. EV was effectiveness of vaccine; CI was confidence interval

As of May 2022, BA.4 and BA.5 have become the main variants in South Africa and are rising rapidly in several European countries. There is also clear evidence that BA.4 and BA.5 encode the same Spike protein, most closely related to BA.2. Four studies reported the neutralizing titers of five booster doses of COVID-19 vaccines against BA.4/5. The random effects model was used because heterogeneity of these studies showed *P* = 0.04, I^2^ = 57.68%. The summary VE of booster vaccination against the BA.4/5 increased dramatically, SMD = 0.77, 95% CI(0.60–0.94), *P* = 0.00 (Fig. 5), and the difference was statistically significant (*P* > 0.05). It was showed that the vaccines would still neutralize BA.4/5.

### Effect of Omicron sublineages on vaccine-induced antibodies susceptibility

Although the booster dosage of COVID-19 vaccines had neutralizing effect against Omicron sublineages, the NT50 of vaccine-induced antibodies declined. We compared the NT50 of vaccine-induced antibodies with control viruses to assess their susceptibility to COVID-19 vaccines. The results revealed that the NT50 in all assays against Omicron sublineages were lower than fivefold compared to the control. The mean fold reduction in susceptibility for BA.1 was 8.7 (2.69–48.7), and the mean NT50 was 519 (8.1–1163) (Figure A and F). The control NT50 was 2642 (250–5493), but the mean NT50 for the BA.1.1 variation was 558 (55–1105), and the mean fold reduction in susceptibility was 5.07 (2.60–7.02) (Figure B and F). The mean NT50 of BA.3 was 609 (216–1111) and fold reduction was 5.0 (3.19–7.47), which were similar with BA.1.1. The mean reduction in BA.2 and BA.2.12.1 sensitivity susceptibility and fold changes decrease were similar, 577 (9.3–1708), 6.3(2.8–12.1) and 579 (315–826), 6.9 (3.2–14.0), respectively. However, the most significant change in NT50 of vaccine-induced antibodies and fold changes of the BA.4/5 was 333 (103–647), 11.7(4.1–20.9).It was a serious hit because the prevalence of BA.4/5 was continuously increasing.

## Discussion

The recently emerged novel coronavirus Omicron sublineages BA.1, BA1.1, BA.2, BA.2.12.1 and BA.4/5, have attracted considerable attention due to their significantly increased heritability [34–36]. In this sublineages, BA.2.12.1 is mainly in the United States, and BA.4/5 is prevalent in South Africa [17]. Several sublineages such as BA.1, BA.2 and BA.3 showed immune escape from neutralizing antibodies [37, 38]. Insufficient data are available on the effectiveness of existing COVID-19 vaccines against Omicron sublineages. Besides, there are a lot of debates about the necessity of the booster dosage vaccines, and the new emerging Omicron sublineages with the increased transmissibility fueling the debates [39]. This study will investigate the roles of COVID-19 booster vaccination in preventing the Omicron infections sublineages and the effect of Omicron sublineages on vaccine-induced antibodies susceptibility.

Through Meta-analysis, we found that the vaccination booster of COVID-19 vaccines against Omicron sublineages had neutralizing effect within a period of time, BA.1/BA.1.1 (SMD = 0.80, 95% CI: 0.75 0.85, *P* = 0.00), BA.2/BA.2.12.1 (SMD = 0.77, 95% CI: 0.69–0.85, *P* = 0.00), BA.3 (SMD = 0.91, 95% CI: 0.83–1.0, *P* = 0.00) and BA.4/5 (SMD = 0.77, 95%CI: 0.60–0.94, *P* = 0.00) (Figs. 2, 3, 4 and 5), whether Moderna or Pfizer, showed greater ability to neutralize many Omicron sublineages, which consistent with previous reports [25, 30, 38]. Although Omicron is less virulent than the Alpha and Delta variants, the transmission of Omicron increased [40], therefore, even with relatively high vaccination rates, Omicron sublineages still cause serious harm worldwide, especially in some high-risk groups [39]. In addition, those who received two dosages of the vaccine and the booster had fewer hospitalizations compared with the severity of illness in the unvaccinated population [41].

**Fig. 6:**
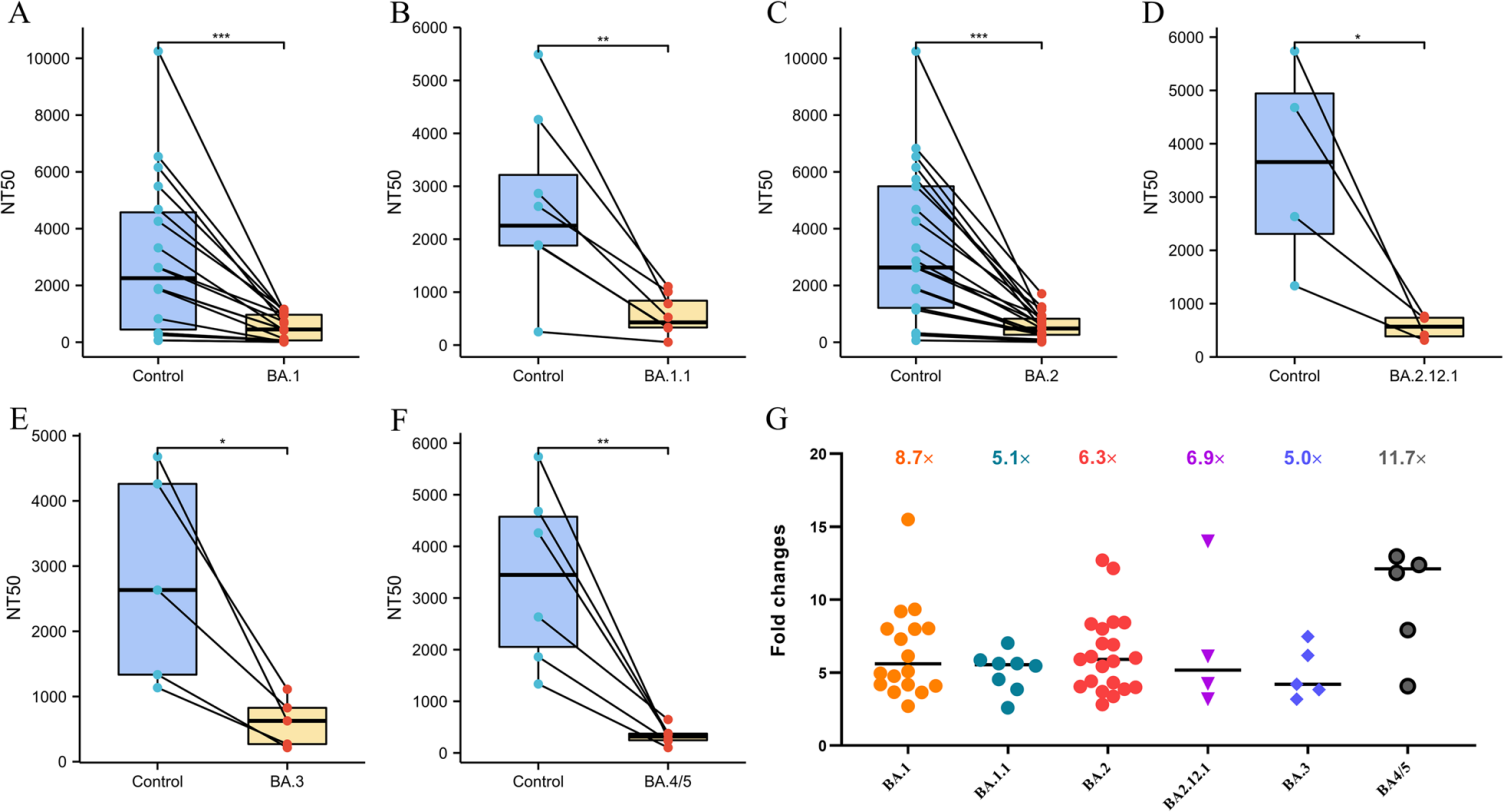
Neutralizing susceptibility for vaccine-induced antibodies against Omicron sublineages. **A**–**E** Neutralizing susceptibility to the Omicron sublineages (BA.1, BA.1.1, BA.2, BA.2.12.1, BA.3, BA.4/5) for vaccine-induced antibodies. Each plot shows the NT50 control virus (on the left) connected by a line to the NT50s of the Omicron BA.1, BA.1.1, BA.2, BA.2.12.1, BA.3, BA.4/5 (on the right). The cyan boxes encompass the interquartile range, the statistical significance of differences between individual groups was assessed by *t* test *p < 0.05, **p < 0.01, ***p < 0.001. **F** Compared with the control group, the reduction of NT50s against each Omicron sublineages, individual points are representative geometric mean fold changes. Bars represent geometric means and error bars represent geometric standard deviations for each group

The booster dosage of COVID-19 vaccines has neutralizing effect on the Omicron sublineages, but its vaccine-induced antibodies NT50 decreased compared with control virus. We evaluated the susceptibility of the vaccines included in the literature and found that all vaccine-induced antibodies showed at least a fivefold reduction in susceptibility. Especially in the case of BA.4/5, the prevalence continued to increase, vaccine effectiveness had most significant reduction, which was more than tenfold (Fig. 6). As the SARS-CoV-2 continues to mutate, new variants will emerge. Although the effectiveness of currently available vaccines against new variants may be reduced, vaccination still provides protection against severe COVID-19 caused by different variants and may reduce the emergence of new variants [2, 42]. Continued attention should be paid to vaccine development in the face of emerging variants in the future.

In our study, we included studies of vaccine neutralization of Omicron Sublinages published between November 26, 2021 and July 28, 2022. We analyzed the degree of reduction in vaccine efficacy and sensitivity by comparing the neutralization titers of vaccine-induced antibodies after the second dose and the booster dose. Our results provide some evidence for evidence-based medicine to empower the public and policy makers. But the study has several limitations. Firstly, the omicron variant appeared in a short period of time, such as BA4/5, and the prevalence of BA3 was relatively low, so there were few related studies, which limited our analysis. In addition, the literatures included in this paper came from different countries, therefore race, age and geographical factors may also bring some heterogeneity.

## Conclusion

In this systematic review and meta-analysis, we discovered that the booster vaccination raised the serum NT50 against SARS-CoV-2 Omicron sublineages, as well as the neutralization ability of vaccine-induced antibodies. However, the vaccine-induced antibodies showed a reduced susceptibility to Omicron sublineages compared with control viruses, especially BA.4/5. Such a reduction in susceptibility of vaccine-induced antibodies could be detrimental to future prevention and treatment of Omicron sublineages infection, therefore some new vaccines need to be investigated.

### Supplementary Information

**Additional file 1**. Search strategy; Figure S1 Risk of bias graph of RCTs; Figure S2 Risk of bias summary of RCTs; Table S1. Risk of bias for included RCTs; Table S2. Risk of bias for included cohort studies

## Data Availability

All data were submitted in the manuscript or supplement material.
