# Supplementary material for: Immune escape of SARS-CoV-2 variants to therapeutic monoclonal antibodies: a system review and meta-analysis
Source: Virol J. 2023 Nov 15;20:266. doi: 10.1186/s12985-023-01977-5 (PMC10652597; doi:10.1186/s12985-023-01977-5)
Supplement: Supplementary file 1 — Additional file 1. Search strategy; Figure S1 Risk of bias graph of RCTs; Figure S2 Risk of bias summary of RCTs; Table S1. Risk of bias for included RCTs; Table S2. Risk of bias for included cohort studies [file 12985_2023_1977_MOESM1_ESM.docx]

**Supplementary Material**

**Search Strategy**

**PubMed (1423 items)**

1. Severe acute respiratory syndrome coronavirus 2 OR SARS-CoV-2 OR coronavirus disease 2019 OR COVID-19;
2. Omicron OR BA.1 OR BA.2 OR BA.3 OR BA.4 OR (BA.5;
3. Vaccine OR vaccines OR (vaccination OR vaccin*;
4. #1 AND #2 AND #3.

**Web of Science (1614 items)**

1: TS= Severe Acute Respiratory Syndrome Coronavirus 2 OR TS=SARS-CoV-2 OR TS=coronavirus disease 2019 OR TS=COVID-19

2: TS= Omicron OR TS=BA.1 OR TS=BA.2 OR TS=BA.3 OR TS=BA.4 OR TS=BA.5;

3: TS=Vaccine OR TS=vaccines OR TS=vaccination OR TS=vaccin*;

4: #1 AND #2 AND #3

**Embase (1989 items)**

1.'severe acute respiratory syndrome coronavirus 2'/exp OR 'sars cov 2':ab,ti OR 'coronavirus disease 2019':ab,ti OR 'covid 19':ab,ti

2.omicron AND ba.1:ab,ti OR ba.2:ab,ti OR ba.3:ab,ti OR ba.4:ab,ti OR ba.5:ab,ti

3.'vaccine'/exp OR vaccine OR vaccines:ab,ti OR vaccination:ab,ti

4.#1 AND #2 AND #3

**Cochrane Library (33 items)**

1.(Severe acute respiratory syndrome coronavirus 2):ti,ab,kw OR (SARS-CoV-2):ti,ab,kw OR (coronavirus disease 2019):ti,ab,kw OR (COVID-19):ti,ab,kw;

2.(Omicron):ti,ab,kw OR (BA.1):ti,ab,kw OR (BA.2):ti,ab,kw OR (BA.3):ti,ab,kw OR (BA.4/5):ti,ab,kw;

3.(Vaccine):ti,ab,kw OR (vaccines):ti,ab,kw OR (vaccination):ti,ab,kw OR (vaccin*) :ti,ab,kw

4.#1 AND #2 AND #3

**Supplementary Figure**

**
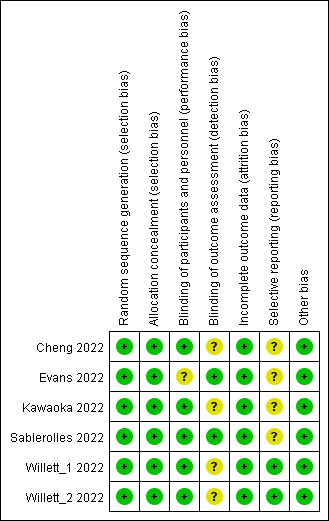

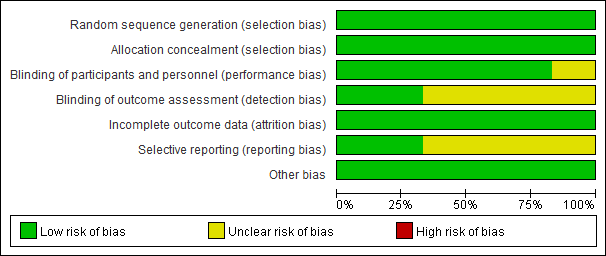
**

**Figure S1 Risk of bias graph of RCTs**

**Figure S2 Risk of bias summary of RCTs**

**Supplementary Tables**

**Table S1. Risk of bias for included RCTs**

| Study (First author) | Randomization process | Allocation concealment | Deviations from intended interventions | Missing outcome data | Measurement of the outcome | Selection of the reported result | Other bias | **Overall bias** |
| --- | --- | --- | --- | --- | --- | --- | --- | --- |
| Cheng 2022 | Low Risk | Low Risk | Low Risk | Unclear Risk | Low Risk | Unclear Risk | Low Risk | Mediate |
| Evans 2022 | Low Risk | Low Risk | Unclear Risk | Low Risk | Low Risk | Unclear Risk | Low Risk | Mediate |
| Kawaoka 2022 | Low Risk | Low Risk | Low Risk | Unclear Risk | Low Risk | Unclear Risk | Low Risk | Mediate |
| Sablerolles 2022 | Low Risk | Low Risk | Unclear Risk | Low Risk | Low Risk | Unclear Risk | Low Risk | Low |
| Willett_1 2022 | Low Risk | Low Risk | Unclear Risk | Unclear Risk | Low Risk | Low Risk | Low Risk | Low - |
| Willett_2 2022 | Low Risk | Low Risk | Unclear Risk | Unclear Risk | Low Risk | Low Risk | Low Risk | Low |

**Table S2. Risk of bias for included cohort studies**

|  | Selection | | | | Comparability | Exposure | | |  |  |
| --- | --- | --- | --- | --- | --- | --- | --- | --- | --- | --- |
|  | Item 1 | Item 2 | Item 3 | Item 4 | Item 5 | Item 6 | Item 7 | Item 8 | Total score | **Quality** |
| Arora 2022 | 1 | 1 | 1 | 1 | 1 | 1 | 0 | 1 | 7 | Good |
| Bowen 2022 | 0 | 1 | 1 | 0 | 1 | 1 | 1 | 0 | 6 | Good |
| Hachmann 2022 | 1 | 1 | 1 | 0 | 1 | 1 | 1 | 1 | 6 | Good |
| Kurhade 2022 | 1 | 1 | 1 | 0 | 1 | 0 | 1 | 1 | 6 | Good |
| Kurhade_2 2022 | 1 | 1 | 1 | 0 | 1 | 0 | 1 | 1 | 6 | Good |
| Lyke 2022 | 0 | 1 | 1 | 0 | 1 | 0 | 1 | 1 | 5 | Moderate |
| Park 2022 | 0 | 1 | 1 | 0 | 0 | 1 | 1 | 1 | 5 | Moderate |
| Pedersen 2022 | 1 | 1 | 1 | 0 | 1 | 1 | 1 | 1 | 7 | Good |
| Tuekprakhon 2022 | 0 | 1 | 1 | 0 | 1 | 0 | 1 | 1 | 5 | Moderate |
| Yu 2022 | 1 | 1 | 1 | 0 | 1 | 1 | 0 | 1 | 6 | Good |
| Zhou_1 2022 | 0 | 0 | 1 | 0 | 1 | 1 | 1 | 1 | 5 | Moderate |
| Zhou_2 2022 | 1 | 0 | 1 | 0 | 1 | 1 | 1 | 1 | 6 | Good |

Note: Item 1: Representativeness of the exposed cohort; item 2: Selection of the non-exposed cohort; item 3: Ascertainment of exposure; item 4: Demonstration that outcome of interest was not present at start of study; item 5: Comparability of cohorts on basis of the design or analysis; item 6: Assessment of outcome; item 7: Was follow up long enough for outcomes to occur; item 8: Adequacy of follow up of cohorts.
